# Identifying the key regulators orchestrating Epstein-Barr virus reactivation

**DOI:** 10.3389/fmicb.2024.1505191

**Published:** 2024-12-05

**Authors:** Yaohao Wang, Jingwen Yu, Yonggang Pei

**Affiliations:** School of Public Health and Emergency Management, Southern University of Science and Technology, Shenzhen, Guangdong, China

**Keywords:** Epstein-Barr virus, virus-host interaction, reactivation, immediate-early proteins, regulation, novel insights

## Abstract

Epstein-Barr virus (EBV) infects more than 90% of the human population worldwide and establishes lifelong infection in hosts by switching between latent and lytic infection. EBV latency can be reactivated under appropriate conditions, leading to expression of the viral lytic genes and production of infectious progeny viruses. EBV reactivation involves crosstalk between various factors and signaling pathways, and the subsequent complicated virus-host interplays determine whether EBV continues to propagate. However, the detailed mechanisms underlying these processes remain unclear. In this review, we summarize the critical factors regulating EBV reactivation and the associated mechanisms. This encompasses the transcription and post-transcriptional regulation of immediate-early (IE) genes, the functions of viral factors on viral DNA replication and progeny virus production, the mechanisms through which viral proteins disrupt and inhibit the host’s innate immune response, and the host factors that modulate EBV reactivation. Finally, we explore the potential applications of novel technologies in studying EBV reactivation, providing novel insights into the investigation of mechanisms governing EBV reactivation and the development of anti-EBV therapeutic strategies.

## 1 Introduction

Epstein-Barr virus (EBV) belongs to the gamma herpesvirus family and is recognized as the first human tumor virus (Epstein, 2012; Damania et al., 2022). It is associated with a variety of human diseases, including infectious mononucleosis (IM), Burkitt lymphoma (BL), Hodgkin’s lymphoma (HL), nasopharyngeal carcinoma (NPC), gastric carcinoma (GC), and multiple sclerosis (MS) (Kutok and Wang, 2006; Damania et al., 2022). EBV primarily initiates infection in the oral epithelial cells, where it then undergoes lytic replication to release progeny virus particles that subsequently infect B lymphocytes. Within these B cells, EBV establishes a latent infection that can be maintained for a lifetime or reactivated to produce new virus particles (Mesri et al., 2014; Scott, 2017). The latent infection and lytic replication of EBV are the keys to its typical life cycle and contribute significantly to its pathogenesis. During latency, EBV establishes one of the four distinct latency patterns (Latency 0, Latency I, Latency II, and Latency III) in B cells, which is represented by the expression of distinct gene sets (Münz, 2019). Meanwhile, the viral genome persists in the host nucleus as a circular extrachromosomal episome. This allows for the establishment of latent infection, as the virus can remain dormant within host cells. The viral episome can anchor to host chromatin via viral antigen EBNA1, and the virus capitalizes on the conditions of host DNA replication to transmit its genome through cell division. The virus remains latent for life within the host and yet a fraction of the virus population still reactivates to enter the lytic cycle.

In contrast, lytic replication is marked by the collective expression of viral genes, the production of infectious progeny viruses, and cell death. EBV lytic replication is initially dependent on the viral immediate-early (IE) proteins Zta and Rta, which are encoded by EBV genes *BZLF1* and *BRLF1*, respectively. These two proteins activate other viral lytic genes and eventually induce lytic replication, among which they are involved not only in DNA replication, protein synthesis, and progeny virus production but also operate with various cellular factors-related signaling pathways (Feederle et al., 2000). Additionally, the genes regulated by Zta and Rta mediate viral immune evasion. Thus, the intervention of EBV reactivation and the subsequent activation of lytic gene functions not only have the potential to limit the transmission of EBV but also offer promising treatment options for EBV-induced malignancies. Furthermore, advancements in various new technologies can provide valuable insights into the critical mechanisms underlying EBV reactivation from multiple perspectives.

To summarize, EBV latency is required for the establishment of a long-term infection, and infectious virus particles are produced during lytic replication. The switch of latency and lytic replication ensures the continuous transmission of EBV infection among the human population. In this review, we summarize the current progress of the identified factors controlling EBV reactivation and provide an overview of how these key factors co-operate to regulate this process. Finally, we highlight the potential applications of novel technologies to further research on EBV reactivation.

## 2 EBV reactivation starts with the expression of viral immediate-early proteins

### 2.1 Zta and Rta initiate EBV reactivation

Epstein-Barr virus reactivation is regulated by various cellular signaling pathways and can be activated under various stimuli, such as hypoxia (Jiang et al., 2006), 12-O-tetradecanoylphorbol-13-acetate (TPA) (Baumann et al., 1998), B-cell receptor (BCR) activation (Murata and Tsurumi, 2014), and reactive oxygen species (ROS) inducers (Yiu et al., 2020). Although how these stimuli initially induce EBV reactivation remains incompletely understood, the cascades following reactivation are triggered uniformly by viral immediate-early proteins Zta and Rta. They are the first two viral proteins actively expressed upon EBV reactivation, while both of them are silenced in EBV latently infected cells (Israel and Kenney, 2003; Kenney and Mertz, 2014). The expression of Zta and Rta marks the onset of the EBV lytic cycle, initiating a multi-stage biological process in which EBV-encoded lytic proteins and various host factors play complex and pivotal roles. Zta and Rta can also activate their promoters (Israel and Kenney, 2003), further boosting the expression of subsequent EBV early and late genes through positive feedback. They serve as transcriptional activators for lytic gene expression by binding to host transcription factors and specific response elements (Urier et al., 1989; Gruffat et al., 1990; Heilmann et al., 2012). Additionally, they are involved in viral genome replication after the onset of reactivation (Rennekamp et al., 2010; El-Guindy et al., 2013). Therefore, the expression of Zta and Rta is essential for the initiation of EBV reactivation, triggering a series of intricate cascading events, including EBV lytic gene expression, viral genome replication, virus particle assembly, and the release of progeny virions.

### 2.2 Transcriptional activity of Zp and Rp is tightly regulated

**FIGURE 1:**
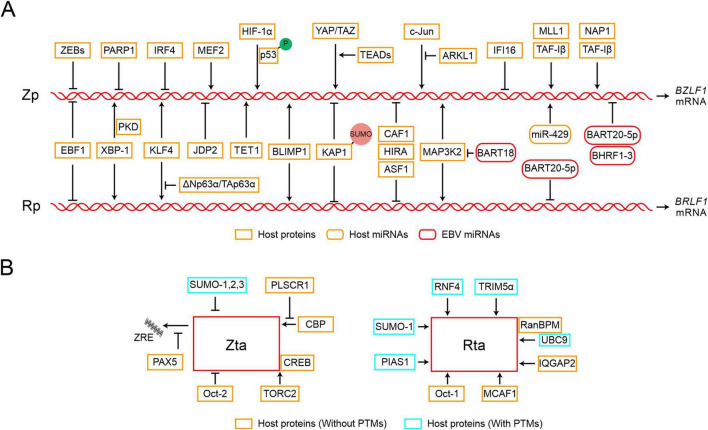
Epstein-Barr virus (EBV) reactivation starts with the expression of viral immediate-early proteins. **(A)** Transcription initiation of *BZLF1* and *BRLF1* is tightly regulated by various factors. The diagram only depicts the regulatory relationships between transcription factors and Zp or Rp and is not related to their regulatory positions. Zp, the promoter of *BZLF1*; Rp, the promoter of *BRLF1*. P, phosphorylation; SUMO, sumoylation. **(B)** The functions of Zta and Rta can be targeted through interactions with specific factors, whether or not post-translational modifications (PTMs) are involved. ZRE, Zta response element.

Given the crucial roles of Zta and Rta in EBV reactivation, transcription factors orchestrate the regulation of *BZLF1* or *BRLF1* transcription by specifically targeting their promoters (Figure 1A and Table 1). Various transcriptional activators and repressors are demonstrated to bind to *BZLF1* and *BRLF1*, whose expression then determines the state of EBV infection (Spitz and Furlong, 2012; Lambert et al., 2018; Pope and Medzhitov, 2018). ZEBs silence the promoter of *BZLF1* (Zp) in a cell-dependent manner, thus promoting the maintenance of viral latency. In particular, ZEB1 directly binds to the Zp, and its overexpression reduces the activity of Zp, suggesting its role in suppressing EBV lytic replication (Feng et al., 2007; Yu et al., 2007). Similar to ZEB1, ZEB2/SIP1 can bind to the same sites on Zp and inhibit *BZLF1* expression to maintain EBV latency (Ellis et al., 2010). PARP1 has also been shown to bind Zp and suppress EBV reactivation, further contributing to cell proliferation and LMP1-mediated oncogenesis (Martin et al., 2016; Lupey-Green et al., 2017; Hulse et al., 2018). Additionally, IRF4 negatively regulates the activity of Zp, while EBF1 negatively regulates the activity of both Zp and Rp (Bristol et al., 2022). In contrast, the MEF2 transcription factor family binds and activates Zp (Murata et al., 2013). XBP-1 can activate Rp independently, whereas the activation of Zp by XBP-1 requires cooperation with PKD (Bhende et al., 2007). KLF4 and BLIMP1 are two differentiation-dependent transcription factors that synergistically induce EBV lytic replication. Among them, KLF4 can bind to and activate both Zp and Rp (Nawandar et al., 2015). BLIMP1 may bind to Rp and indirectly induce transcription of Zp and Rp (Reusch et al., 2015). KLF4 and BLIMP1 also synergistically activate LMP1, which promotes lytic replication in EBV-infected epithelial cells by enhancing the expression of Zta and Rta (Nawandar et al., 2017). HIF-1α directly binds to Zp, not Rp, to induce EBV reactivation, which requires phosphorylated p53 as a co-activator for the induction of the *BZLF1* gene (Kraus et al., 2017; Kraus et al., 2020). The Hippo signaling effectors YAP and TAZ interact with Zp in epithelial cells to activate *BZLF1* expression, and TEADs enhance the activation of YAP/TAZ on Zp (Van Sciver et al., 2021a). In epithelial cells, ΔNp63α maintains EBV latency, while in Burkitt lymphoma, TAp63α restrains the activation of EBV. ΔNp63α can block the activation of Zp which is mediated by KLF4 and Rta protein (Van Sciver et al., 2021b). The transcriptional activator C-Jun binds to and activates Zp (Flemington and Speck, 1990; Liang et al., 2002), whereas ARKL1 interacts with C-Jun, inhibiting the activity of C-Jun and negatively regulating EBV reactivation. The interaction between ARKL1 and C-Jun is mediated by the CK2 kinase-regulating subunit CK2β (Siddiqi et al., 2019). KAP1 is also an important factor in the maintenance of viral latency, whose sumoylation binds to the lytic promoters and suppresses their transcriptional activity (Bentz et al., 2015). Additionally, IFI16 and KAP1 function together to silence *BZLF1* expression (Xu et al., 2022). In summary, these cellular factors can directly bind to Zp and Rp or recruit other factors for binding, thereby modulating their transcription activity and initiating subsequent processes in EBV reactivation.

**TABLE 1:** Summary of the cellular factors that regulate the expression or functions of Zta and Rta.

Factors	Functions	References
ZEB1	Interact with the Zp and suppresses the Zp activation	Feng et al., 2007; Yu et al., 2007; Ellis et al., 2010
ZEB2		
PARP1	Interact with Zp and stabilize CTCF	Lupey-Green et al., 2017; Lupey-Green et al., 2018
IRF4	Negatively regulate Zp	Bristol et al., 2022
EBF1	Negatively regulate Zp and Rp	
MEF2	Bind and activate the Zp	Murata et al., 2013
XBP-1	Activate Rp and activate Zp cooperatively with activated PKD	Bhende et al., 2007
KLF4	Combine and activate Zp as well as Rp, activate the promoter of LMP1	Nawandar et al., 2015
BLIMP1	Bind Rp to activate lytic replication, activate the promoter of LMP1	Reusch et al., 2015
HIF-1α	Binding Zp and phosphorylation of p53 is necessary	Kraus et al., 2017; Kraus et al., 2020
YAP	Zp interacts with YAP and TAZ TEADs promote Zp activation by YAP/TAZ	Van Sciver et al., 2021a
TAZ		
TEADs		
ΔNp63α	Restrict the activation of Zp and Rp by KLF4	Van Sciver et al., 2021b
ARKL1	Suppress the activity of c-Jun	Siddiqi et al., 2019
KAP1	Collaborate to block EBV reactivation	Bentz et al., 2015
IFI16		Xu et al., 2022
TAF-Iβ	TAF-Iβ recruits MLL1 and is involved in chromatin modification of Zp NAP1 cooperates with TAF-Iβ to promote EBV reactivation	Mansouri et al., 2013
NAP1		
CAF1	Promote H3K27me3 on IE promoter	Zhang et al., 2020
HIRA		
ASF1		
JDP2	Bind to the ZII element and recruit the HDAC3	Murata et al., 2011
miR-429	Target Zp and promote BCR-mediated reactivation	Lin et al., 2010
miR-BHRF1-3	Target the 3′UTR of *BZLF1*	Fachko et al., 2022
miR-BART20-5p	Target the 3′UTR of both *BZLF1* and *BRLF1*	Jung et al., 2014; Kim et al., 2016
miR-BART18	Target the 3′UTR of MAP3K2	Qiu and Thorley-Lawson, 2014
PLSCR1	Block Zta-CBP transcriptional activity	Kusano and Ikeda, 2019
TORC2	Interact with Zta-CREB to activate Zp	Murata et al., 2009
PAX5	Block Zta binds to the ZRE	Raver et al., 2013
IQGAP2	Participate in the activation of Rp	Lin et al., 2023
Oct-1	Cooperative induction of Rp activation with Rta	Robinson et al., 2011
Oct-2	Inhibit Zta feature	Robinson et al., 2012
MCAF1	Promote the formation of the Zta-MCAF1-Rta and Sp1-MCAF1-Rta complex	Chang et al., 2005; Chang et al., 2010
RanBPM	Interact with SUMO-E2 (Ubc9) to promote Rta sumoylation	Chang et al., 2008
SUMO-1, 2, 3	Inhibit Zta transactivation and increased Rta transactivation	Chang et al., 2004; Hagemeier et al., 2010
RNF4	Rta Ubiquitination	Yang et al., 2013
TRIM5α		Huang et al., 2017
PIAS1	Enhance Rta-mediated EBV lytic activation, inhibit Zta-mediated Zp activation	Zhang et al., 2017

The transcription of Zp and Rp is also regulated by epigenetic modifications. Zp and Rp are heavily methylated and silenced in latent infection, whereas histone acetylation of Zp and Rp enhances gene accessibility and promotes the binding of transcription factors during lytic replication (Speck et al., 1997; Miller et al., 2007; Woellmer and Hammerschmidt, 2013; Daskalogianni et al., 2015; Guo and Gewurz, 2022). In epithelial cells, TAF-Iβ recruits host methyltransferase MLL1 to promote H3K4 dimethylation and H4K8 acetylation, leading to the chromatin modifications of Zp region and the following EBV reactivation (Mansouri et al., 2013). CAF1, HIRA, and ASF1 are host factors that cooperatively load histone H3 and H4 onto the EBV genome, inhibiting the activity of IE promoter mediated by H3K27me3 and thus hindering lytic reactivation. Depletion of CAF1 protein induces EBV reactivation in Burkitt lymphoma cells (Zhang et al., 2020). JDP2 has been shown to bind to the Z II element of Zp and recruit HDAC3, reducing histone acetylation on the promoter and inhibiting EBV reactivation (Murata et al., 2011). The host factor C-Jun facilitates the binding of TET1 to Zp, enhancing the demethylation of Zp and activating *BZLF1* expression (Zhang et al., 2016). When these inhibitory histone modifications are relieved, the transcription factors can bind to the promoters of lytic genes and activate EBV lytic replication (Jung et al., 2007; Murata et al., 2012; Guo and Gewurz, 2022). DNA methylation is not always detrimental to EBV lytic replication, for instance, Zta can specifically bind to the methyl-Zta responsive element (meZRE) and activate lytic gene promoters in a methylation-dependent manner, thereby disrupting epigenetic silencing (Bergbauer et al., 2010; Kalla et al., 2012; Almohammed et al., 2018). Furthermore, Zp and Rp can be targeted by various miRNAs. Following BCR stimulation in plasma cells, the increasing miR-429 disrupts EBV latency by targeting Zp (Lin et al., 2010). EBV miR-BHRF1-3 targets the 3′UTR region of *BZLF1* gene and attenuates Zta expression (Fachko et al., 2022). Similarly, EBV miR-BART20-5p binds to the 3′ UTR region of these two IE genes, resulting in transcription inhibition (Jung et al., 2014). EBV miR-BART18 targets the 3′ UTR of MAP3K2 mRNA, affecting the expression of both *BZLF1* and *BRLF1* (Qiu and Thorley-Lawson, 2014).

Therefore, the activity of IE promoter, Zp and Rp, is modulated by transcriptional regulatory factors and epigenetic modifications, which collectively control EBV reactivation through regulating the expression of IE genes.

### 2.3 Host factors target Zta and Rta to regulate EBV reactivation

In addition to the transcription activity, the host proteins interact with Zta or Rta and modulate their capabilities in EBV reactivation (Figure 1B and Table 1). PLSCR1 can directly interact with Zta, antagonize the interaction between Zta and CBP, and further block the transcriptional activity of the Zta-CBP complex (Kusano and Ikeda, 2019). In Zta-mediated transcriptional activation, TORC2 acts as a co-activator and interacts with the Zta-CREB complex to activate Zp transcription, which involves the calcineurin-TORC pathway (Murata et al., 2009). PAX5 interacts directly with Zta and inhibits Zta-mediated lytic gene expression (Raver et al., 2013). PAX5 can also bind to viral TRs (tandem terminal repeats) and regulate the expression of viral genes (Arvey et al., 2012; Lee et al., 2015; Lee et al., 2016). IQGAP2 co-localizes with Rta in the nucleus and participates in the activation of Rp (Lin et al., 2023). Oct-1 and Oct-2 induce EBV reactivation through interactions with Rta and Zta, respectively. In particular, Oct-1 synergistically induces viral reactivation through direct interaction with Rta, which is independent of Oct-1 binding with DNA (Robinson et al., 2011). In contrast, Oct-2 negatively regulates virus reactivation by inhibiting the functions of Zta protein (Robinson et al., 2012). MCAF1 serves as an interaction partner for both Zta and Rta, facilitating their synergistic activation. The interaction between MCAF1 and Rta promotes the formation of Sp1-MCAF1-Rta complex at the Sp1 binding site, which ultimately results in the activation of Rp (Chang et al., 2005). In addition, the Zta-MCAF1-Rta complex also binds to the Zta response element (ZRE) and synergistically promotes the expression of EBV lytic genes (Chang et al., 2010). RanBPM simultaneously interacts with SUMO-E2 enzyme Ubc9 and Rta, which promotes SUMO-1-mediated Rta sumoylation and the transactivation activity of Rta (Chang et al., 2008).

Post-translational modifications (PTMs), such as phosphorylation, ubiquitination, sumoylation, and other covalent modifications are capable of regulating the associated activity of Zta or Rta (Walsh et al., 2005). The phosphorylation of Zta and Rta has been shown to affect the binding of Zta to DNA, and the transactivation capabilities of both Zta and Rta (Francis et al., 1999; Bhende et al., 2005; Asai et al., 2006). Furthermore, four lysine residues on Zta are targets of ubiquitination, and the ubiquitination of Zta is essential for progeny production in the viral lytic replication (Zhao et al., 2020). All SUMO isoforms 1, 2, and 3 can induce Zta sumoylation at the lysine residue 12 (Murata et al., 2010). Although sumoylation does not alter Zta localization, it inhibits the transactivation ability of Zta and the Zta-mediated lytic cycle (Hagemeier et al., 2010). In contrast, Rta transactivation activity is enhanced during SUMO-1-mediated sumoylation (Chang et al., 2004). Two E3 ubiquitin ligases, RNF4 and TRIM5α, interact with Rta and promote its ubiquitination, restricting viral lytic replication (Yang et al., 2013; Huang et al., 2017). RNF4 can target SUMO-2-conjugated Rta and knockouts of both RNF4 and TRIM5α increase Rta expression. PIAS1 enhances Rta-mediated activation of EBV lytic genes via sumoylation, but PIAS1 inhibits Zta and C/EBP β-mediated Zp activation, and this inhibition does not rely on its SUMO ligase activity (Zhang et al., 2017). Therefore, the host proteins interact with viral IE proteins and modulate their activities, potentially depending on the post-translational modifications, which play crucial roles in EBV reactivation.

## 3 EBV reactivation is subject to multiple layers of regulation after initiation

### 3.1 EBV-encoded proteins function in lytic replication

**FIGURE 2:**
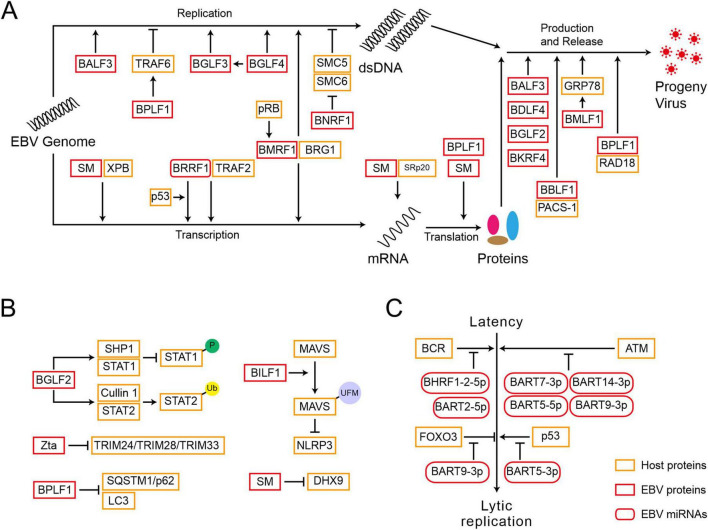
Epstein-Barr virus reactivation is subject to multiple layers of regulation after initiation. **(A)** EBV-encoded proteins function in the lytic gene expression, viral DNA replication, and progeny virus production. **(B)** EBV-encoded proteins disrupt the host’s innate immune response to promote reactivation. P, phosphorylation; Ub, ubiquitination; UFM, UFMylation. **(C)** EBV-encoded miRNAs are crucial for reactivation.

The expression of EBV lytic genes determines the progression of a lytic cycle (Figure 2A and Table 2). During viral DNA replication, EBV-encoded proteins not only regulate the replication of viral genome but also hijack or antagonize host factors to control host DNA replication. BGLF4, an EBV-encoded protein kinase, not only activates the expression of BGLF3, but together they control post-replication processes and regulate the expression of late genes (El-Guindy et al., 2014). BGLF4 also phosphorylates UXT at the threonine-3 site, weakening the interaction between UTX and p65, which represses NF-κB-mediated *BZLF1* and *BRLF1* expression and promotes the viral lytic cycle (Chang et al., 2012). EBV-encoded nuclear protein SM is essential for lytic gene expression. SM binds to and stabilizes mRNA, modulating the splicing of cellular and viral pre-mRNAs, which depends on the interaction between SM and the SR splicing factor, specifically SRp20 (Verma et al., 2010). Additionally, as a transcription activator, SM binds to and recruits cellular XPB (xeroderma pigmentosum group B-complementing protein, a subunit of TFII H), subsequently targeting the promoters of late genes (Verma et al., 2020). SM also binds to CBC, eIF4F, and PABP, thereby enhancing the translation initiation of mRNPs, which are involved in protein synthesis (Mure et al., 2018). BRRF1 interacts with TRAF2 and induces lytic gene expression in a TRAF2-dependent manner. BRRF1 also co-activates lytic replication with p53. In the absence of p53, BRRF1-mediated activation of lytic genes is significantly reduced (Hagemeier et al., 2011).

**TABLE 2:** Epstein-Barr virus (EBV)-encoded factors are involved in the reactivation.

Factors	Function	References
BGLF4	Regulation of late gene expression with BGLF3	El-Guindy et al., 2014
	Downregulation of NF-κB-mediated inhibition of BZLF1 and BRLF1	Chang et al., 2012
SM	EBV pre-mRNA splicing	Verma et al., 2010
	Recruit XPB to target late gene promoters	Verma et al., 2020
	Enhancing the translation initiation of mRNPs	Mure et al., 2018
	Antagonize the antiviral function of DHX9	Fu et al., 2019
BRRF1	Synergize with p53 to induce lytic	Hagemeier et al., 2011
BMRF1	Synergize with BRG1 to promote related gene activation	Su et al., 2017
	Regulated by the host factor pRB	Myers et al., 2023
BPLF1	Deubiquitinate TRAF6	Saito et al., 2013
	Promote EBNA1 mRNA translation	Liu et al., 2023
	Promote the production of infectious viruses with RAD18	Kumar et al., 2014
	Deubiquitinate TOP2	Li et al., 2021
	Attenuate autophagy	Ylä-Anttila et al., 2021
BNRF1	Mediate degradation of SMC5/6 complex	Yiu et al., 2022
BALF3	DNA synthesis, packaging, and release of progeny viruses	Chiu et al., 2014
BBLF1	Promote progeny virus production and release through PACS-1	Chiu et al., 2012; Uddin et al., 2023
BDLF4	Late gene expression and progeny virus production	Watanabe et al., 2015; Sato et al., 2019
BGLF2	Enhanced BZLF1 expression and progeny virus release	Liu and Cohen, 2016
	Antagonize IFN antiviral response	Jangra et al., 2021
BMLF1	Activate ATF6 and GRP78	Chen L. -W. et al., 2021
BKRF4	Generation of infectious progeny viruses	Masud et al., 2017
BILF1	Block activation of NLRP3 inflammasome	Yiu et al., 2023
BZLF1	Disrupt TRIM24/TRIM28/TRIM33 complex	De La Cruz-Herrera et al., 2023
miR-BART5-5p	Coordinately regulates ATM, miR-BART9-3p target and inhibit FOXO3	Lung et al., 2018; Chen Y. et al., 2021
miR-BART7-3p		
miR-BART9-3p		
miR-BART14-3p		
miR-BART5-3p	Inhibit p53	Zheng et al., 2018
miR-BHRF1-2-5p	Attenuate BCR-mediated reactivation	Chen et al., 2019
miR-BART2-5p		

The cofactor of viral DNA polymerase, BMRF1, is a key protein for lytic DNA replication. BMRF1 promotes the activation of BcLF1 and BLLF1 through interacting with the transcription regulator BRG1 (Su et al., 2017). The cellular pRb facilitates lytic reactivation, whose deletion inhibits lytic replication and BMRF1 expression in differentiated epithelial cells (Myers et al., 2023). The host factor TRAF6, once activated by polyubiquitin chain conjugation, binds to LMP1 and mediates NF-κB signaling, which inhibits the viral lytic cycle. However, EBV-encoded BPLF1 interacts with and deubiquitinates TRAF6, thereby inhibiting the LMP1-mediated NF-κB pathway and promoting viral DNA replication (Saito et al., 2013). Meanwhile, BPLF1 promotes the translation of EBNA1 mRNA (Liu et al., 2023). The host SMC5/6 complex recognizes and occupies R-loops, which are essential for DNA double-strand separation and the loading of core replication proteins during the lytic cycle, inhibiting the synthesis of EBV genome (Yiu et al., 2022). EBV-encoded BNRF1 membrane protein degrades the SMC5/6 complex through calpain proteolysis and the E3 ubiquitin ligase Cullin-7 dependent proteasome degradation pathway, allowing EBV to escape host restriction on viral genome replication and the production of infectious progeny virions (Yiu et al., 2022). Therefore, EBV-encoded proteins are key regulators in controlling the processes of viral lytic replication.

### 3.2 EBV-encoded proteins play critical roles in virus assembly and progeny production

After the EBV genome completes replication, the virus synthesizes capsid proteins and assembles progeny virus particles within the host cell, ultimately releasing infectious virus particles to infect other cells (Figure 2A and Table 2). EBV-encoded BALF3 is involved not only in viral DNA synthesis but also in the packaging and release of mature virus particles (Chiu et al., 2014). BBLF1 is a myristoylated and palmitoylated protein, whose myristoylation enhances its stability and facilitates its membrane anchoring in the trans-Golgi network (TGN). Through interaction with PACS-1, a cytoplasmic protein, BBLF1 mediates retrograde transport from endosomes to the TGN and supports the production of progeny viruses (Chiu et al., 2012). The NED region of BBLF1 is key to the release of mature virus particles (Uddin et al., 2023). BDLF4, a viral protein expressed early in viral lysis, is a component of the lytic gene regulatory complex. BDLF4 knockout severely restricts the expression of late genes and reduces the production of progeny virus (Watanabe et al., 2015). By hijacking the host CDK2 complex, BDLF4 is phosphorylated at multiple amino acid residues, preventing its degradation through ubiquitin-mediated pathways. Among these residues, the phosphorylation of Thr-91 is critical for maintaining BDLF4 stability (Sato et al., 2019). BGLF2 enhances Zta expression in both B cells and gastric cancer cells, promoting the release of progeny viruses through the p38 signaling pathway. In contrast, its absence impairs reactivation triggered by inducers (Liu and Cohen, 2016). BMLF1 activates the UPR (unfolded protein response) sensor ATF6 by initiating the proteolytic cleavage, which transcriptionally activates the GRP78 promoter, a central regulator in the UPR, via ER stress response elements. Although the deletion of GRP78 does not affect the expression of EBV cleavage genes, it reduces the production of viral particles (Chen L. -W. et al., 2021). BKRF4, a late lytic protein, has a limited impact on viral gene replication and protein expression, yet it is crucial for the production of infectious progeny viruses (Masud et al., 2017). BPLF1 modulates the DNA damage response induced by EBV lytic replication, collaborating with RAD18 to enhance the production of infectious viruses (Kumar et al., 2014). BPLF1 also deubiquitinates TOP2, inhibiting the DNA damage response induced by TOP2 and promoting viral production (Li et al., 2021). These EBV-encoded proteins are responsible for regulating virus assembly and progeny production by modulating various cellular factors.

### 3.3 EBV-encoded proteins disrupt the host’s innate immune response to promote reactivation

To successfully replicate and propagate within the infected cells, EBV has evolved various strategies to evade the host’s innate immune surveillance (Figure 2B and Table 2). EBV-encoded BGLF2 is an essential factor to counteract the interferon (IFN)-mediated antiviral response. BGLF2 can recruit the SHP1 phosphatase to STAT1 and inhibit the phosphorylation of STAT1. Additionally, BGLF2 recruits STAT2 to the E3 ubiquitin ligase Cullin 1, leading to its ubiquitination and degradation (Jangra et al., 2021). EBV G-protein-coupled receptor BILF1 recruits the E3 ligase UFL1 (UFM1 specific ligase 1) to facilitate the interaction between MAVS and UFM1 (ubiquitin fold modifier 1), leading to the MAVS UFMylation. This process results in MAVS degradation via the mitochondrial-lysosomal pathway, thus blocking the activation of NLRP3 inflammasomes (Yiu et al., 2023). EBV-encoded SM protein inhibits the transcriptional activation of DHX9, counteracting its antiviral function and reducing the effects of host immunity during reactivation (Fu et al., 2019). Cellular RNA Helicase DHX9 enhances the antiviral innate immunity in the host, limiting the production of EBV viral particles. TRIM24 and TRIM33 are cellular antiviral factors that inhibit *BZLF1* expression, however, Zta interacts with these two proteins in EBV reactivation, leading to the destruction of the TRIM24-TRIM28-TRIM33 complex and the degradation of TRIM24 and TRIM33 (De La Cruz-Herrera et al., 2023). Autophagy impairs virus replication and transmission, but BPLF1 targets and inhibits the recruitment of selective autophagy receptor SQSTM1/p62 to LC3, reducing the adverse effects of autophagy on the lytic cycle (Ylä-Anttila et al., 2021).

In conclusion, the replication of the EBV genome, the production of progeny viruses, and the host’s immune response induced by various factors constitute an interconnected regulatory network. The balance among these components ultimately determines the progression following reactivation.

### 3.4 EBV-encoded miRNAs are crucial for reactivation

In addition to the EBV-encoded proteins described above, the process of EBV reactivation is also regulated by distinct miRNAs (Figure 2C and Table 2). EBV miR-BART20-5p blocks BAD-induced Caspase-3-dependent cellular apoptosis, thus contributing to the maintenance of EBV latency (Kim et al., 2016). Four EBV miR-BARTs (BART5-5p, BART7-3p, BART9-3p, and BART14-3p) cooperatively regulate and suppress ATM, resulting in the disruption of EBV reactivation (Lung et al., 2018). Another EBV miR-BART5-3p maintains latency by suppressing p53 expression (Zheng et al., 2018). EBV miR-BART9-3p, along with its homologous miR-141-3p, targets and inhibits the expression of the host transcription factor FOXO3 during BCR cross-linking, thereby reducing its inhibitory effect on BCR-induced viral lytic activation (Chen Y. et al., 2021). In contrast, EBV miR-BHRF1-2-5p, miR-BART2-5p, and the host miR-17-5p have been shown to weaken the BCR-mediated reactivation of EBV *in vivo* (Chen et al., 2019).

## 4 Host factors determine the progress of EBV reactivation

**FIGURE 3:**
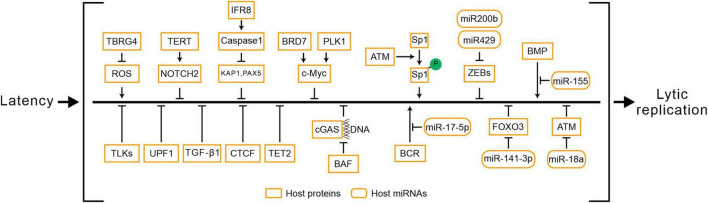
Host factors determine the progress of EBV reactivation. P, phosphorylation.

Epstein-Barr virus reactivation involves the crosstalk of multiple signaling pathways, and the processes are also regulated by various host factors (Figure 3 and Table 3). The abundance of Myc protein serves as a checkpoint switch for EBV lytic replication (Guo et al., 2020), and cytokines regulate EBV reactivation by modulating *Myc* expression (Biswas et al., 2021; Li et al., 2023). BRD7 binds with EBNA1 and co-localizes in the promoter of *c-Myc* to regulate *c-Myc* transcriptional activation, furthermore, BRD7 deficiency activates the Myc-mediated lytic cycle (Li et al., 2023). PLK1 may restrict EBV reactivation through the regulation of *c-Myc* activity, and inhibition of PLK1 promotes EBV reactivation (Biswas et al., 2021). ROS has been shown to function as a stimulator for lytic EBV reactivation (Yiu et al., 2020), while TBRG4 reduces ROS generation in host cells and inhibits EBV lytic replication (Zhang et al., 2022). TERT maintains EBV latency through the NOTCH2/BATF pathway. Firstly, the expression of TERT is correlated with BATF and NOTCH2. Secondly, TERT activates the NOTCH promoter in a dose-dependent manner (Giunco et al., 2015). During lytic induction, IRF8 enhances Caspase expression and activation (Lv et al., 2018), which subsequently destabilizes EBV reactivation suppressors such as KAP1 (Bentz et al., 2015; Xu et al., 2022), PAX5 (Arvey et al., 2012; Raver et al., 2013; Lee et al., 2015; Lee et al., 2016). Another family of host factors, TLKs, plays a role in the reactivation of EBV during its latent phase, and the knockout of TLKs leads to EBV reactivation (Dillon et al., 2013). BAF restricts the binding of nuclear DNA to cGAS and suppresses its activity to promote EBV reactivation (Guey et al., 2020; Broussard et al., 2023). UPF1 mediates the nonsense-mediated decay of BRLF1 transcripts, controlling the reactivation of EBV (van Gent et al., 2021). In Burkitt lymphoma cell lines, TGF-β1 is shown to activate EBV lytic replication (di Renzo et al., 1994). In NPC cells, EBV lytic infection induces ATM activation. Subsequently, the phosphorylation of Sp1 at the serine-101 residue mediated by ATM activation contributes to the formation of viral replication compartment (Hau et al., 2015).

**TABLE 3:** Host factors can modulate EBV reactivation.

Factors	Function	References
BRD7	Regulate c-Myc	Li et al., 2023
PLK1		Biswas et al., 2021
TBRG4	Reduce ROS production	Zhang et al., 2022
TERT	Activate the NOTCH promoter	Giunco et al., 2015
IRF8	Destruction KAP1 and PAX5 through Caspase	Lv et al., 2018
TLKs	Block EBV reactivation	Dillon et al., 2013
BAF	Limit the binding of nuclear DNA and cGAS	Guey et al., 2020
UPF1	Degradation of BRLF1 transcripts	van Gent et al., 2021
TGF-β1	Activates EBV lytic replication	di Renzo et al., 1994
ATM	Involved in the formation of viral replication compartments	Hau et al., 2015
miR-18a	Reactivate EBV and cause genomic instability	Cao et al., 2018
miR200b	Downregulate ZEBs expression	Ellis-Connell et al., 2010
miR429		
miR-155	Block BMP-mediated reactivation	Yin et al., 2010
miR-141-3p	Target and inhibit FOXO3	Chen Y. et al., 2021
miR-17-5p	Attenuate BCR-mediated reactivation	Chen et al., 2019
CTCF	Combine with the EBV genome to sustain latency	Lupey-Green et al., 2018
TET1	Activate BZLF1 expression	Zhang et al., 2016
TET2	Modulate the EBV DNA methylation	Lu et al., 2017

Furthermore, the host factors regulate EBV reactivation via epigenetic modifications (Pei et al., 2020). After hypoxia-induced DNA damage response in B cells, ATM activation suppresses EBV lytic gene expression and reduces viral load. The host miR-18a suppresses the ATM-mediated DNA damage response in an EBV-dependent manner, leading to EBV reactivation and host genomic instability (Cao et al., 2018). Host miR-200b and miR-429 induce viral lytic replication and the production of infectious progeny viruses by downregulating ZEBs expression (Ellis-Connell et al., 2010). Another host miR-155 restricts BMP-mediated EBV reactivation (Yin et al., 2010). Extensive methylation of the viral genome in latency leads to the silencing of lytic genes (Woellmer and Hammerschmidt, 2013; Germi et al., 2016). The host factor TET2, in cooperation with EBNA2, modulates the DNA methylation status of EBV, and the knockout of TET2 promotes the expression of lytic genes (Lu et al., 2017). Additionally, CTCF maintains EBV latency by binding to the viral genome, while PARP1 promotes CTCF expression and stabilizes CTCF binding, which facilitates the maintenance of EBV latency (Lupey-Green et al., 2018). In summary, the reactivation of EBV is a complex process involving the interplay of multiple mechanisms, regulated by a diverse range of host factors and miRNAs (Supplementary Table 1).

## 5 Novel insights to explore the mechanisms of EBV reactivation

The emergence of numerous innovative technologies and methodologies enables a deeper understanding of the mechanisms underlying EBV latency and reactivation. First of all, Alphafold3 is a powerful tool capable of predicting the structures of complexes involving proteins and nucleic acids, despite the limitation of predicting only static structures (Hsu et al., 2014). Utilizing Alphafold3 to model the known host factors and their binding sites, we could unveil identical or similar structural domains in these factors when bound to Zp and Rp. This approach can guide the development of specific small-molecule drugs through virtual screening. Additionally, Alphafold3 can be harnessed to predict the structures of lesser-known EBV gene products, providing valuable insights into their functions and interactions with the host.

Secondly, the rapid development in Natural Language Processing (NLP) also brings novel insights for biomedical research. Multiple pre-trained models, like BioGPT (Luo et al., 2022), have enabled us to access the biomedical literature with higher accuracy and availability. Based on the abstract texts of 21 million articles, researchers mapped the entire biomedical literature landscape onto a two-dimensional (2D) plot, enabling available cross-referencing of biomedical metadata (González-Márquez et al., 2024). Concerning EBV infection, we have developed a functional encyclopedia of EBV-related cellular pathways and signaling networks from 36,105 EBV-relevant scientific publications (Yu et al., 2024). NLP methods have also been shown to elucidate molecular regulatory pathways. reguloGPT is developed to successfully infer the m6A-specific knowledge graph and demonstrate its utility in elucidating m6A’s regulatory mechanisms across various cancer phenotypes (Wu et al., 2024). Applications of the NLP techniques bear high potential in the assistance of unveiling new targets for EBV reactivation.

Thirdly, the emerging gene editing technologies will greatly expand our capacity to elucidate virus-host interactions and develop targeted antiviral strategies. For instance, CRISPR interference (CRISPRi) and CRISPR activation (CRISPRa) are endogenous gene regulation systems derived from the CRISPR/Cas9 platform (Tian et al., 2019; Tian et al., 2021). Both techniques mediate gene expression without inducing double-strand breaks (DSBs), thereby preserving the genomic sequence. In the context of gene function studies, CRISPRa-mediated transcriptional activation overcomes the limitations of cDNA overexpression. CRISPRi and CRISPRa can also be employed to modulate non-coding RNAs (ncRNAs). Thus, genome-wide CRISPRi/a screens in EBV studies may facilitate the discovery of additional ncRNAs associated with viral latency and lytic cycle regulation. Furthermore, another gene editing technology, base editors, can mediate conversions among the four DNA bases without inducing DSBs (Anzalone et al., 2020). Given that EBV encodes several critical oncogenic and enzymatic proteins that regulate its life cycle, the base editor screens may assist in identifying key functional domains and sites within these proteins, thereby advancing both basic research and drug development efforts. Furthermore, the EBV genome exists as circular episomes within the host cells, and isolating these episomes has posed a significant limitation in functional studies. Recent studies have successfully isolated human extrachromosomal DNAs using CRISPR-CATCH (Hung et al., 2022). By further refining and optimizing this technique, we may be able to isolate EBV episomes for subsequent studies on the biological functions.

Finally, the combined use of multiple omics approaches enables a comprehensive understanding of the impact of EBV infection on host cells and the key mechanisms underlying virus infection. For instance, a combined analysis of metabolomics and transcriptomics has identified IDO1 as a critical host factor for establishing EBV latency in B cells; inhibiting IDO1 disrupts EBV-induced B cell transformation (Müller-Durovic et al., 2024). Integrating metabolomics with proteomics may reveal additional vital metabolic pathways altered by EBV infection. Consequently, the approved metabolic regulators could be used to modulate human metabolic pathways, alleviating symptoms associated with EBV-associated diseases. The concurrent application of various omics technologies with CRISPR techniques also aids in elucidating the key mechanisms of EBV infection. For example, the integration of metabolomics with CRISPR system has demonstrated that methionine metabolism is a significant pathway for maintaining EBV latency (Guo et al., 2022). By leveraging a combination of techniques, we can gain a deeper understanding of the complex interactions between EBV and its host, thereby guiding the development of novel therapeutic strategies.

The emerging technologies mentioned above, including structural prediction tools, literature data mining tools, and genome editing tools give us access to an unprecedented amount of data to screen for critical factors underlying EBV reactivation. For instance, CRISPRi using the lytic model will produce thousands of essential target genes for EBV reactivation. Multimodal analysis combining RNA-seq will reveal the functional key regulators. Furthermore, mass spectrometry combined with protein-protein docking screen enables the identification of co-regulators and the discovery of molecular mechanisms. Despite these rapid technological innovations, the complexity of biological systems, particularly the intricate and diverse machinery involved in virus-host interactions, still requires careful experimentation and thorough evaluation to dissect and unveil the nuanced mechanisms.

## 6 Conclusion

Epstein-Barr virus is widespread across the globe and is associated with a variety of human diseases. EBV is almost impossible to be cleared from the host due to its effective latency and periodic reactivation that is initiated by appropriate signals. Development of an effective druggable target to mitigate EBV reactivation bears an important therapeutic opportunity for curing EBV-related diseases. Inhibiting the lytic genes is also an effective means to treat EBV infections. However, discovering the ultimate cure for EBV-induced diseases remains a significant challenge. In this review, we summarize the key factors governing EBV reactivation, including factors involved in regulating IE gene expression, the functions of IE gene products, viral replication, the release of progeny virions, and the innate immune response triggered by viral infection. In conclusion, given the abundance of cellular regulatory elements, the critical factors may be still hiding beneath cloaks and silently puppeteering the pathogenic events through unknown mechanisms. Integrating established virological research methods with novel technologies could facilitate the identification of key regulators in the EBV reactivation, and ultimately eradicate the negative effects of EBV on human well-being.
