# Supplementary material for: Identifying the key regulators orchestrating Epstein-Barr virus reactivation
Source: Front Microbiol. 2024 Dec 5;15:1505191. doi: 10.3389/fmicb.2024.1505191 (PMC11655498; doi:10.3389/fmicb.2024.1505191)
Supplement: Supplementary file 1 [file Table_1.docx]

**Table S1. The abbreviations or primary functions for host and viral factors are listed in alphabetical order.**

| Abbreviation | Definition or primary functions |
| --- | --- |
| ARKL1 | Arkadia-like 1 |
| ASF1 | Anti-silencing factor 1 |
| ATF6 | Activating transcription factor 6 |
| ATM | Ataxia telangiectasia mutated |
| BAF | Barrier-to-autointegration factor 1 |
| BALF3 | Tripartite terminase subunit 1 |
| BBLF1 | Cytoplasmic envelopment protein 3 |
| BDLF4 | Late gene expression regulator |
| BGLF2 | Cytoplasmic envelopment protein 2 |
| BGLF4 | Serine/threonine-protein kinase |
| BILF1 | G-protein coupled receptor |
| BKRF4 | Tegument protein |
| BLIMP1 | B-lymphocyte-induced maturation protein 1 |
| BMLF1 | EBV protein SM |
| BMP | Bone morphogenetic protein |
| BMRF1 | DNA polymerase processivity factor |
| BNRF1 | Major tegument protein |
| BPLF1 | Large tegument protein deneddylase |
| BRD7 | Bromodomain-containing protein 7 |
| BRG1 | Brahma-related gene 1 |
| BRLF1 | Replication and transcription activator |
| BRRF1 | Transcriptional activator |
| BZLF1 | Lytic switch protein |
| CAF1 | Chromatin assembly factor 1 |
| cGAS | Cyclic GMP-AMP synthase |
| c-Jun | Transcription factor AP-1 subunit Jun |
| CTCF | CCCTC-binding factor |
| DHX9 | DExH-box helicase 9 |
| DNMT3A | DNA methyltransferase 3 alpha |
| EBF1 | Early B-cell factor 1 |
| FOXO3 | Forkhead Box O3 |
| GRP78 | 78-kDa glucose-regulated protein |
| HDAC3 | Histone Deacetylase 3 |
| HIF-1α | Hypoxia-inducible factor 1-alpha |
| HIRA | Histone regulatory homologue A |
| IFI16 | Interferon-gamma inducible protein 16 |
| IQGAP2 | IQ motif containing GTPase activating protein 2 |
| IRF4 | Interferon regulatory factor 4 |
| IRF8 | Interferon regulatory factor 8 |
| JDP2 | Jun dimerization protein 2 |
| KAP1 | KRAB-associated protein 1 |
| KLF4 | Krueppel-like factor 4 |
| LMP1 | Latent membrane protein 1 |
| MAP3K2 | Mitogen-activated protein kinase kinase kinase 2 |
| MCAF1 | MBD1-containing chromatin-associated factor 1 |
| MEF2 | Myocyte enhancer factor 2 |
| MLL1 | Lysine methyltransferase 2A |
| mRNPs | Messenger ribonucleoparticles |
| NAP1 | Nucleosome assembly protein 1 |
| NLRP3 | NLR family pyrin domain containing 3 |
| NOTCH | Neurogenic locus notch homolog protein |
| Oct-1 | Octamer-binding transcription factor 1 |
| Oct-2 | Octamer-binding transcription factor 2 |
| PACS-1 | Phosphofurin acidic cluster sorting protein 1 |
| PARP1 | Poly (ADP-ribose) polymerase 1 |
| PAX5 | B-cell-specific transcription factor |
| PIAS1 | Protein inhibitor of activated STAT protein 1 |
| PKD | Protein kinase D |
| PLK1 | Polo Like kinase 1 |
| PLSCR1 | Phospholipid scramblase 1 |
| pRB | Retinoblastoma-associated protein |
| RAD18 | RING-type E3 ubiquitin transferase |
| RanBPM | Ran-binding protein in microtubule-organizing center |
| RNF4 | Ring-finger protein 4 |
| SMC5/6 | Structural maintenance of chromosomes protein 5/6 |
| SP1 | Transcription factor |
| SRp20 | Pre-mRNA splicing factor |
| SUMO | Small ubiquitin-like protein |
| TAF-Iβ | Template-activating factor Iβ |
| TAZ | WWTR1, WW domain containing transcription regulator 1 |
| TBRG4 | Transforming growth factor-beta regulator 4 |
| TEAD | TEA domain transcription factor |
| TERT | Telomerase reverse transcriptase |
| TET1 | Ten-eleven translocation 1 |
| TET2 | Ten-eleven translocation 2 |
| TGF-β1 | Transforming growth factor beta 1 |
| TLKs | Tousled-like kinases |
| TOP2 | DNA topoisomerase II |
| TORC2 | Transducer of CREB protein 2 |
| TRAF6 | TNF receptor-associated factor 6 |
| TRIM24 | Tripartite motif-containing protein 24 |
| TRIM28 | Tripartite motif-containing protein 28 |
| TRIM33 | Tripartite motif-containing protein 33 |
| TRIM5α | Tripartite motif-containing protein 5 alpha |
| UPF1 | Nonsense mRNA reducing factor 1 |
| XBP-1 | X-box-binding protein 1 |
| XPB | Xeroderma pigmentosum group B-complementing protein |
| YAP | Yes-associated protein |
| ZEB1 | Zinc finger E-box binding homeobox 1 |
| ZEB2 | Zinc finger E-box-binding homeobox 2 |
| ΔNp63α | N-terminal truncated isoform of p63 |
